# Identification of proteins associated with bast fiber growth of ramie by differential proteomic analysis

**DOI:** 10.1186/s12864-021-08195-9

**Published:** 2021-12-02

**Authors:** Fu Li, Zheng Zeng, Renyan Huang, Yanzhou Wang, Touming Liu

**Affiliations:** 1 Institute of Bast Fiber Crops, Chinese Academy of Agricultural Sciences, Changsha, 410205 China; 2 Hunan Institute of Plant Protection, Changsha, 410205 China

**Keywords:** Ramie, fiber growth, Secondary wall biosynthesis, Proteome, Differential expression

## Abstract

**Background:**

Ramie is an important fiber-producing crop in China, and its fibers are widely used as textile materials. Fibers contain specialized secondary cellular walls that are mainly composed of cellulose, hemicelluloses, and lignin. Understanding the mechanism underlying the secondary wall biosynthesis of fibers will benefit the improvement of fiber yield and quality in ramie.

**Results:**

Here, we performed a proteomic analysis of the bark from the top and middle parts of the stem, where fiber growth is at different stages. We identified 6971 non-redundant proteins from bast bark. Proteomic comparison revealed 983 proteins with differential expression between the two bark types. Of these 983 proteins, 46 were identified as the homolog of known secondary wall biosynthetic proteins of *Arabidopsis*, indicating that they were potentially associated with fiber growth. Then, we proposed a molecular model for the secondary wall biosynthesis of ramie fiber. Furthermore, interaction analysis of 46 candidate proteins revealed two interacting networks that consisted of eight cellulose biosynthetic enzymes and seven lignin biosynthetic proteins, respectively.

**Conclusion:**

This study sheds light on the proteomic basis underlying bast fiber growth in ramie, and the identification of many candidates associated with fiber growth provides important basis for understanding the fiber growth in this crop.

**Supplementary Information:**

The online version contains supplementary material available at 10.1186/s12864-021-08195-9.

## Background

Fibers, that are widespread among vascular plants and are present in various organs, provide mechanical support to the organs and the plant body, and thereby, they essential for plant growth and development. In addition, plant fibers are important for humans because they are major sources of raw materials in the production of paper, textiles, and composites, as well as the structural components of timber and energy-rich components of wood fuel. Plant fibers contain specialized secondary cellular walls that are mainly composed of cellulose, hemicelluloses (xylan and glucomannan), and lignin^1^. Accordingly, fiber growth is mainly involved in secondary wall biosynthesis. For example, mutation of *Arabidopsis* cellulose synthase (CesA) genes causes a severe reduction in cellulose content and secondary wall thickening, and consequently its xylem fibers are unable to support the erect phenotype [1], suggesting that the biosynthesis is crucial for the fiber growth,

Currently, In *Arabidopsis*, hundreds of genes participate in the biosynthesis of secondary walls, and they are co-ordinately turned on temporally and spatially, which is controlled by a transcriptional network [1, 2]. In this regulatory network, NAC transcription factors, such as SND1, NST1, and NST2, are the top-level master switches that are capable of triggering the entire secondary wall biosynthetic program; MYB46 and its close homolog MYB83, act as the second-level master switches regulating secondary wall biosynthesis; then, MYB46/MYB83 can activate a battery of downstream transcription factors, including NAC regulators SND2 and SND3, and MYB transcriptional factors MYB20, MYB42, MYB43, MYB52, MYB54, MYB58, MYB63, MYB69, MYB85, MYB103 [1]. Therefore, in Arabidopsis, secondary wall biosynthesis is modulated by a NAC-MYB-based regulatory network in which NAC and MYB transcriptional factors showed a level-by-level regulation to precisely control this growth process.

Ramie (*Boehmeria nivea* L. Gaud) is one of the world’s oldest fiber crops and has been cultivated for thousands of years in China [3]. Ramie fibers are extracted from stem bark and possess many excellent characteristics, including smooth texture, high tensile strength, and long fiber strands, and its length can rarely reach 55 cm [4]. However, their use in textiles is associated with defects, including confined elasticity, elongation potential, and resistance to dyeing. Thus, focusing on the developmental process will help to improve the yield and quality of ramie fibers. In the past decade, hundreds of genes potentially related to fiber development have been detected in ramie using homologous and/or expression analysis [5–8]. Furthermore, large numbers of non-coding RNAs (ncRNAs) play a role in fiber growth of ramie [9, 10], suggesting that complex regulation underlies the fiber growth. These studies provided important basis for exploring the regulation of fiber growth in ramie. However, an exact mechanism underlying the fiber formation and development remains largely unknown in this crop.

Previously, the microscopic examination of stem bark has revealed that bast fibers from different parts of the stem are distinctly different at the developmental level, that is, the bark at the top part of the stem (TPS) does not initiate fiber growth, whereas bark at the middle part of the stem (MPS) has a large number of fibers whose secondary walls are thickened (Additional file 1: Fig. S1) [7]. In this study, we characterized and compared the proteome of the bark at the TPS and MPS, thereby to identify of proteins associated with fiber growth in ramie.

## Methods

### Experimental material, tissue sampling, and protein extraction

The elite cultivar Zhongzhu 1 was planted in the experimental farm of the Institute of Bast Fiber Crops, Chinese Academy of Agricultural Sciences, Yuanjiang, China, in June 2016. As shown in Fig. S1, the TPS and MPS bark were separately collected from the individual of Zhongzhu 1 (30 days old), namely, the 10 cm-length bark collected from below the midpoint of stem was used as the MPS sample, whereas the bark that was away from the top of stem from 10 cm to 20 cm was used as the TPS sample. The collected samples were immediately frozen in liquid nitrogen. Three replicates were sampled for the TPS and MPS bark; and for each replicate, the phloem barks of TPS and MPS were collected from a same individual.

The sample powder was ground with liquid nitrogen and sonicated in lysis buffer. Then, an equal volume of Tris-saturated phenol was added, and the mixture was vortexed. After centrifugation, the proteins were precipitated using ammonium sulfate saturation. Finally, the protein was re-dissolved in 8 M urea and the protein concentration was determined with a BCA kit according to the manufacturer’s instructions.

### Proteome UHPLC-MS/MS analysis

Prior to performing UHPLC-MS/MS analysis, the extracted proteins were treated as follows. First, the proteins were digested using trypsin, according to the method described by Ye et al. [11]. Next, the tryptic peptides of each sample were labelled using tandem mass tags (TMT) using a TMT kit according to the manufacturer’s protocol, and then fractionated by high pH reverse-phase HPLC using a Thermo Betasil C18 column. These peptides were further used for proteomic analysis.

Proteome analysis for the fractionated peptides was performed using an LC-MS/MS system. Briefly, these peptides were separated by dissolving them in 0.1% formic acid (solvent A) using an EASY-nLC 1000 UPLC system. Subsequently, the peptides were subjected to a nanospray ionization source followed by tandem mass spectrometry (MS/MS) in Q Exactive™ Plus (Thermo) coupled online to the UPLC with the following parameters: scan range, 350–1800 m/z and resolution, 70,000. Peptides were selected for MS/MS using normalized collision energy with a resolution of 17,500. A data-dependent procedure that alternated between one MS scan followed by 20 MS/MS scans with 15.0 s dynamic exclusion was applied. Automatic gain control was set at 5 × 10^− 4^. The fixed first mass was set as 100 m/z.

### Database search and data analysis

The MS/MS data from the proteome analysis were processed using the program Maxquant (v.1.5.2.8) [12]. Tandem mass spectra were searched against the protein sequences annotated from the genome of ramie [13]. The following parameters were specified in the protein database searches: only tryptic peptides with up to two missed cleavage sites were permitted; 20 ppm (first search) and 5 ppm (main search) mass tolerances for MS and 0.6 Da for MS/MS fragment ions; carbamidomethyl on cysteine as a fixed modification; and protein N-acetylation, oxidized methionine, and phospho_STY (serine, threonine, and tyrosine) were permitted as variable modifications [11]. The FDR was adjusted to < 1%. Only proteins with at least two peptides including at least one unique peptide were reserved for further analysis.

Subsequently, the normalization of protein abundance was achieved in two steps. First, the abundance of spike-in samples (quantified by TMT) was used to adjust the abundance among three replicates. Second, each TMT quantification channel within an individual replicate was normalized based on the total reporter ion intensity. The Pearson correlation test was used to evaluate the reproducibility of the three replicates. The fold-change in protein abundance was calculated as the ratio of normalized protein abundance between the MPS and TPS. A protein with a change of greater than two-fold was defined as a significantly differentially expressed protein (*P* < 0.05).

### Parallel reaction monitoring (PRM) analysis

PRM technology is a highly efficient tool for quantifying the expressed abundance of target proteins [14]. To verify the differences of protein abundance identified from UHPLC-MS/MS analysis, nine proteins were further quantified by PRM analysis. Briefly, after finishing the protein extraction and trypsin digestion, peptide mixture was loaded onto a PicoFrit capillary column (Woburn, MA, USA) packed with ReproSil-Pur Basic C18 reverse-phase resin, and was separated in an EASY-nLC 1000 UPLC system. Then, the eluate was examined via mass spectrometry using Q Exactive™ Plus (Thermo Fisher Scientific), which was coupled to the UPLC online. After a full-scan event, the MS/MS scans in PRM mode were triggered for target proteins. The set parameters were the same as those of the proteome UHPLC-MS/MS analysis. Three biological replicates were performed.

### Bioinformatics analysis

The functions of proteins identified from the proteome analysis were annotated by searching against three public databases, including Gene Ontology (GO), InterPro, and the Kyoto Encyclopedia of Genes and Genomes databases, with a default parameter. The subcellular localization of proteins was predicted using the software package WoLF PSORT [15]. The enrichment of GO functional categories for differentially expressed proteins was analyzed using GOseq, which is based on the Wallenius non-central hypergeometric distribution. A *P*-value less than 0.01 was identified as a significant enrichment. Protein–protein interactions were analyzed by searching proteins against the STRING database [16]. Only interactions between the proteins belonging to the searched dataset were selected, thereby excluding external candidates; protein pairs with a confidence score of more than 0.7 were defined as an interaction.

### Orthologous analysis of protein

Orthologous analysis was carried out between the differentially expressed proteins and known secondary wall biosynthetic proteins of *Arabidopsis* using the bidirectional best hit method [17]. There are 13 NAC and 16 MYB proteins involved in the regulation of secondary wall biosynthesis in *Arabidopsis* [1]. These NAC/MYB protein sequences, along with all KNOX proteins of *Arabidopsis*, were downloaded from the PlantTFDB database [18]. Then, these *Arabidopsis* proteins, together with ramie proteins, were subjected to phylogenetic analysis. Briefly, sequence alignment was carried out using the Clustal program [19], and an unrooted phylogenetic tree was constructed using MEGA 5 using the Neighbor-Joining (NJ) method and the bootstrap test was carried out with 1000 replicates [20].

## Results

### Characterization of the proteome of stem barks

**Fig. 1:**
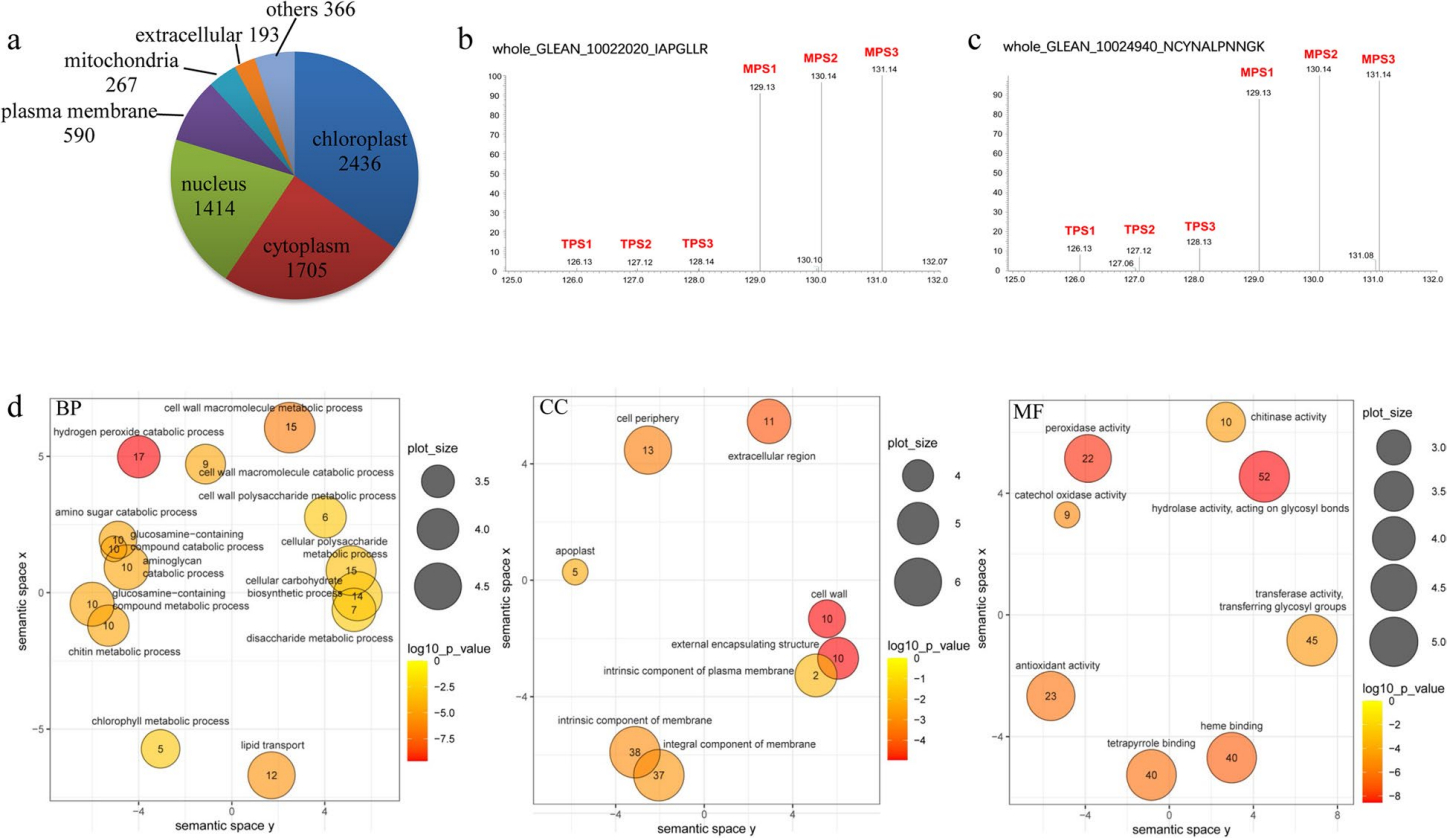
Characteristics of the proteome of bast barks in ramie. **a** Subcellular localizations of all 6971 identified proteins based on the prediction by Gene Ontology (GO) annotation. Numbers in the graph represent the number of proteins located in the corresponding organelle. The secondary MS/MS spectra of whole_GLEAN_10022020 (**b**) and whole_GLEAN_10024940 (**c**) were quantified in the bark from the top and middle part of the stem, where the fibers do not initiate growth or are growing, respectively. The x and y axis in the figure of (**b**) and (**c**) indicated the mass-to-charge ratio (m/z) and the relative abundance of peptide, respectively. **d** GO enrichment analyses. GO terms were significantly enriched for the following three GO categories: biological process, cellular component, and molecular function. Each circle stands for a GO term. Within the Cartesian coordinates (x, y), the closer the circles, the more the GO terms are related. The measurement of the circles is relative to the number of related GO terms. The numbers in the circles show the counts of differentially expressed proteins in the GO terms. The color of the circle represents the significance of the enrichment (*P* < 0.01)

To understand the protein profiling underlying fiber growth in ramie, we used the UHPLC-MS/MS system to analyze the proteome of the bast barks from the TPS and MPS, generating ~ 0.45 million spectra (Table 1). After analyzing the spectra of the proteome, 45,052 peptides representing 6971 non-redundant individual proteins were identified from the bast bark. To obtain an overview of the function of proteins identified from the proteome, we performed a bioinformatics prediction of their subcellular localization, and revealed that most of the proteins were distributed in the chloroplast, cytoplasm, and nucleus (Fig. 1a), accounting for 79.7% of the identified proteins.

**Table 1:** Summary of proteome analysis in the stem barks of ramie

Item	Number
Total spectrum	447,621
Peptides	45,052
Proteins	6971
Differential proteins	983
Up-regulated proteins	533
Down-regulated proteins	450

### Differentially expressed proteins between the barks of the TPS and MPS

There was good repeatability in parallel replicates from the datasets (Additional file 1: Fig. S2). Thus, these datasets were further used to detect differential proteins in the bast bark of the TPS and MPS. Finally, 983 proteins were identified with significant differences in the peptide abundance in the two investigated bast barks (*P* < 0.05; Additional file 2: Table S1), among which 14 showed a difference in the expression of more than 10-fold (Table 2). Compared with the TPS, there were 533 and 450 proteins whose expression was up- and downregulated in the MPS, respectively. whole_GLEAN_10022020 and whole_GLEAN_10024940 were the homologs of caffeic acid 3-O-methyltransferase (COMT) and peroxidase 2, two key enzyme for lignin biosynthesis and polymerization in *Arabidopsis* [21, 22], and they displayed the largest expressed change among these differentially expressed proteins, with a 55.6-fold and 20.4-fold increase in the protein abundance in the fiber-developing barks, respectively (Fig. 1b, c).

**Table 2:** Proteins with more than 10-fold expression change in the fiber developmental barks of ramie stem

Protein	Abundance in TPS	Abundance in MPS	Fold	Direction	*P* value	Annotation
whole_GLEAN_10022020	0.04	1.97	55.6	Up	2.8E-03	peroxidase 2
whole_GLEAN_10024940	0.08	1.62	20.4	Up	5.6E-05	caffeic acid 3-O-methyltransferase
whole_GLEAN_10023456	0.10	1.84	18.5	Up	1.0E-03	gibberellin-regulated protein 1
whole_GLEAN_10010427	0.10	1.73	17.9	Up	5.0E-04	MLP-like protein 328
whole_GLEAN_10011096	0.09	1.61	17.2	Up	1.7E-05	ferredoxin, root R-B1-like
whole_GLEAN_10022053	0.11	1.89	16.7	Up	1.7E-05	protein EXORDIUM-like 2
whole_GLEAN_10012318	0.14	1.87	13.3	Up	1.8E-04	SODA protein
whole_GLEAN_10010430	0.14	1.86	13.0	Up	2.6E-04	MLP-like protein 328
whole_GLEAN_10019453	0.15	1.81	12.5	Up	3.7E-03	Cysteine-rich receptor-like protein kinase 29
whole_GLEAN_10017104	0.15	1.80	12.2	Up	1.0E-06	GDSL esterase/lipase 1
whole_GLEAN_10029752	0.15	1.85	12.0	Up	1.3E-03	peamaclein
whole_GLEAN_10025111	0.16	1.84	11.4	Up	3.9E-06	Sugar transport protein 13
whole_GLEAN_10013709	1.83	0.17	10.8	Down	2.6E-09	GDSL esterase/lipase
whole_GLEAN_10029185	1.73	0.16	11.0	Down	7.1E-07	putative aquaporin NIP5-1

TPS and MPS indicated the bark sample collected from the top and middle part of stems

To obtain an overview of differentially expressed proteins, we performed GO enrichment analysis. Our results revealed that the differentially expressed proteins were markedly enriched in cell wall growth-related GO terms (Fig. 1d; Additional file 1: Table S2), including “cell wall,” “cell wall macromolecule metabolic process,” “cell wall macromolecule catabolic process,” “cellular polysaccharide metabolic process,” “cell wall polysaccharide metabolic process,” and “cellular carbohydrate biosynthetic process.” Because fiber formation mainly requires the growth and thickening of the secondary cellular wall, our results indicate that these enriched proteins have roles in the fiber formation of ramie.

### Validation of expressed differences by PRM analysis

To verify the expressed difference identified proteome analysis, the expressed abundance of nine proteins that are homologs of *Arabidopsis* secondary wall biosynthetic proteins (including three CesA, one SUS protein, and the ortholog of *Arabidopsis* PAL1, AtPRX2, COMT, CAD3, and CslA9) were quantified in the barks of the TPS and MPS by PRM analysis. Our results further validated their expressed differences in two examined tissues, and found that their fold-changes in the MPS, detected by PRM analysis, were larger than those detected by proteome analysis (Table 3). Because the proteins analyzed by PRM technology were chosen randomly, PRM analysis suggested that the differences in candidate proteins from the proteome were reliable.

**Table 3:** Validation of the expression change for nine candidate proteins potentially involved in the fiber growth using the PRM analysis

Protein ID	Abundance in TPS	Abundance in MPS	Fold	*P* value	Fold in proteome	Annotation
whole_GLEAN_10027761	1.90	0.10	0.05	6.5E-06	0.35	Cellulose synthase
whole_GLEAN_10016752	1.75	0.25	0.15	2.0E-05	0.37	Cellulose synthase
whole_GLEAN_10005256	1.66	0.34	0.20	1.5E-04	0.32	Cellulose synthase
whole_GLEAN_10017913	0.33	1.67	4.99	1.4E-05	2.51	Sucrose synthase
whole_GLEAN_10017970	0.25	1.75	7.00	9.9E-06	3.34	caffeic acid 3-O-methyltransferase COMT
whole_GLEAN_10005908	0.33	1.67	5.00	4.0E-06	2.40	Cinnamyl alcohol dehydrogenase CAD
whole_GLEAN_10022662	1.41	0.59	0.42	1.3E-04	0.50	Phenylalanine ammonia-lyase
whole_GLEAN_10022017	0.01	1.99	320.65	3.4E-06	6.90	Peroxidase 2
whole_GLEAN_10022522	1.96	0.04	0.02	4.5E-04	0.21	Glucomannan 4-beta-mannosyltransferase 9

TPS and MPS indicated the bark sample collected from the top and middle part of stems

### Candidate proteins for secondary wall biosynthesis in ramie fibers

**Fig. 2:**
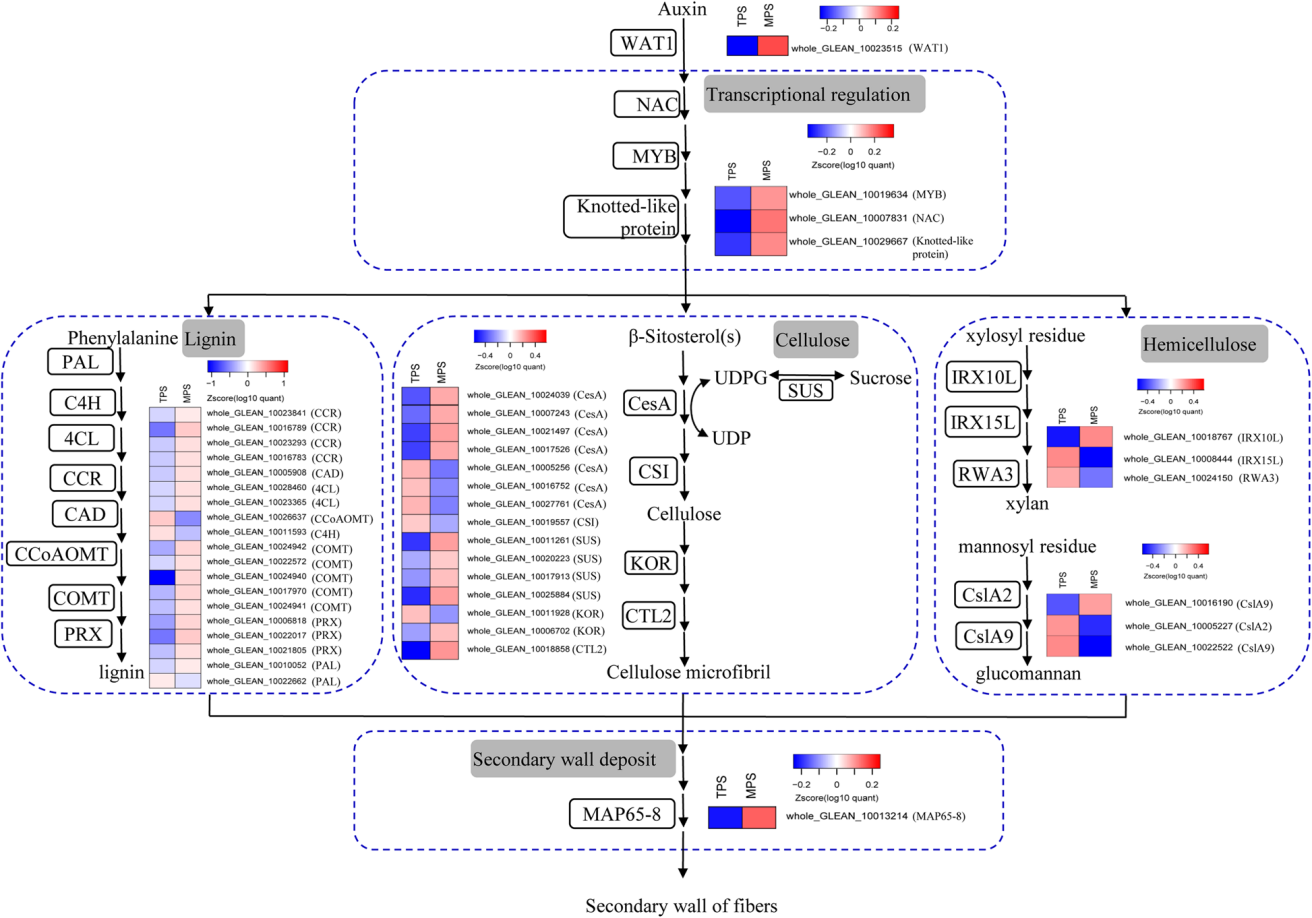
Proposed model for the secondary wall biosynthesis of ramie fibers. In total, 46 candidate proteins were classified into five functional categories, including transcriptional regulation, lignin, cellulose, and hemicellulose biosynthesis, as well as secondary wall deposition. The box to the left of the arrows in each block indicates the family of candidate proteins. The heatmap in each block represents the expression difference of candidate proteins belonging to corresponding functional categories. The full name of the enzyme in each box can be found in the Additional file 1: Table S3

Thickened secondary walls are mainly composed of cellulose, hemicelluloses, and lignin, and are one of the characteristic cellular components of sclerenchyma cells, such as fiber cells. Therefore, fiber growth is mainly involved in the biosynthesis and deposition of secondary walls. In this study, based on the orthologous analysis of *Arabidopsis* secondary wall biosynthetic proteins, 42 differentially expressed proteins were identified as potentially being involved in the biosynthesis and assembly of cellulose/hemicelluloses/lignin (Additional file 1: Table S3). Thus, we proposed a model for the secondary wall biosynthesis of ramie fibers (Fig. 2).

Cellulose is biosynthesized by the catalysis of essential enzymes, including cellulose synthase (CesA) [23] and sucrose synthase (SUS) [24, 25]. Then, it is further assembled and deposited in the microfibril by proteins, such as KORRIGAN (KOR) [26] and chitinase-like protein 2 (CTL2) [27]. In this study, we identified seven CesAs, one CesA-interactive protein (involved in the transport of the CesA complex), and four SUSs that showed considerable differences in the peptide abundance in the fiber developmental barks (Fig. 2). Additionally, two KOR homologs and one CTL2 homolog also showed an expression difference between the two bark tissues investigated, suggesting that they play roles in the deposition of cellulose.

Lignin is the second most common component of the secondary wall of plant fibers, and there are many enzymes identified to catalyze lignin biosynthesis and polymerization in *Arabidopsis*, such as cinnamoyl-CoA reductase (CCR), COMT, 4-coumarate-CoA ligase (4CL), lignin-forming anionic peroxidase (PRX), and phenylalanine ammonia-lyase (PAL) [21, 22]. In this study, we identified 20 differentially expressed proteins that are homologs of *Arabidopsis* lignin biosynthetic enzymes, including four CCRs, five COMTs, two 4CLs, four PRXs, and two PALs (Fig. 2). Interestingly, we observed the AtPRX2 homolog whole_GLEAN_10022020 showed scarce expression in the TPS, but displayed high protein abundance in the fiber developmental barks.

Xylan and glucomannan are two common hemicelluloses found in the secondary wall of plant fibers. In this study, three differentially expressed proteins were identified as homologs of *Arabidopsis* xylan biosynthetic proteins. i.e., IRX10L [28], IRX15L [29], and RWA3 [30]. Additionally, three ramie homologs of CslA2 and CslA9, which are involved in the biosynthesis of glucomannan [31], showed distinct differences in their expression abundance in the TPS and MPS (Fig. 2).

After finishing the biosynthesis of cellulose, hemicelluloses, and lignin, they must be further assembled and deposited into the secondary walls of fibers, in which many proteins are involved, such as the 65 kDa microtubule-associated protein 8 (MAP65–8), which is a key protein for microtubule bundling [32]. In this study, the expression of whole_GLEAN_10013214, a homolog of MAP65–8 in ramie, was upregulated during the fiber developmental stage in the MPS (Fig. 2).

### Candidate regulators for the secondary wall biosynthesis of fibers

**Fig. 3:**
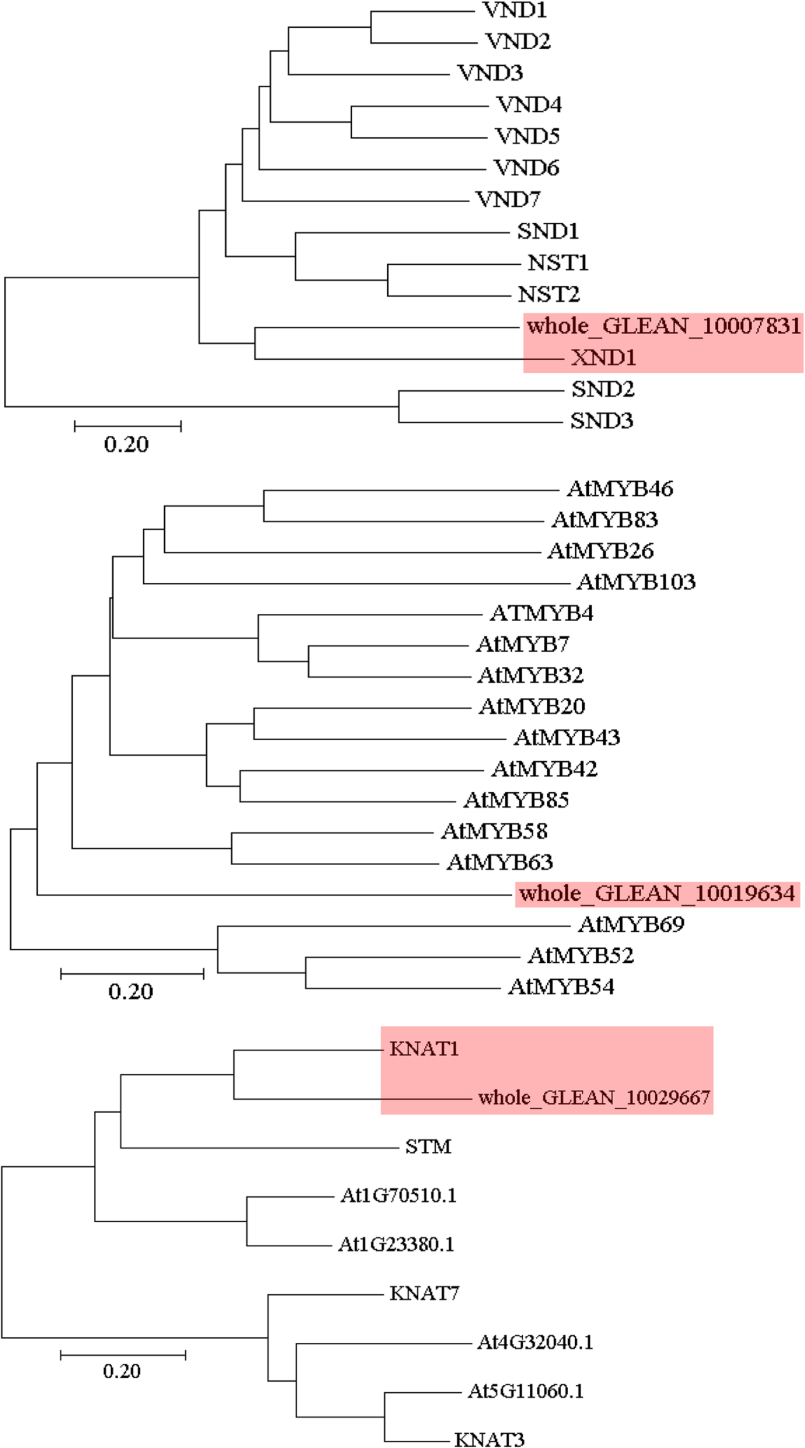
Phylogenetic tree of candidate transcription regulators and *Arabidopsis* proteins. *Arabidopsis* NAC and MYB regulators with known functions involved in the secondary wall biosynthesis and all *Arabidopsis* KNOX proteins were used. The unrooted tree was generated using the MEGA 5 program with the Neighbor-Joining method. Bootstrap values from 1000 replicates are indicated at each node

In *Arabidopsis*, auxin is a key hormone in triggering the biosynthesis ofsecondary wall, and WAT1, a vacuolar auxin transporter, is essential for fiber differentiation and secondary wall thickening [33]. In this study, the expression of one WAT1 homolog, whole_GLEAN_10023515, displayed distinct change in the fiber developmental barks. In addition, the abundance of three transcription factors (i.e., MYB protein whole_GLEAN_10019634, NAC protein whole_GLEAN_10007831, and KNOX protein whole_GLEAN_10029667) markedly increased in the MPS (Fig. 2; Additional file 1: Table S3), indicating their potential roles in the regulating of the secondary wall biosynthesis. Because a mass of MYB and NAC proteins is involved in secondary wall biosynthesis in *Arabidopsis* [1], we investigated the sequence similarity between these two differentially expressed NAC/MYB proteins and known *Arabidopsis* secondary wall biosynthesis-regulated NAC/MYB proteins, and discovered that whole_GLEAN_10007831 was the ortholog of XND1 (Fig. 3), a key regulator of secondary wall biosynthesis [34]. The ortholog of whole_GLEAN_10019634 was not included in the known secondary wall biosynthesis-regulated MYB proteins of *Arabidopsis*. In addition, the sequence similarities between whole_GLEAN_10029667 and *Arabidopsis* KNOX proteins were investigated, whereby the former was found to be an ortholog of KNAT1 that is associated with fiber number [35].

### In silico interaction analysis among candidate proteins

**Fig. 4:**
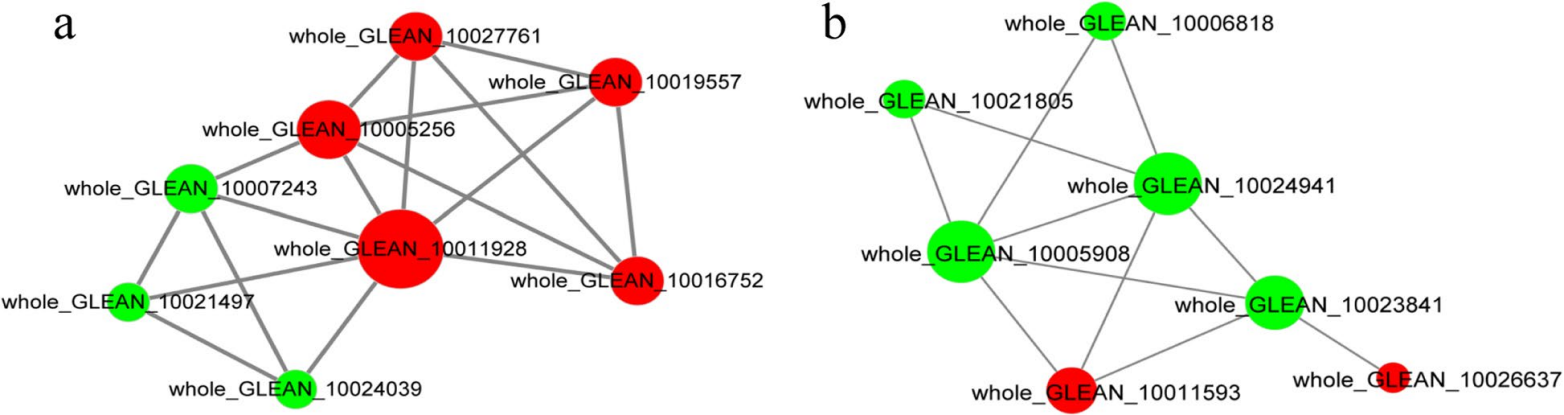
Interaction networks comprised of eight cellulose biosynthetic enzymes (**a**) and seven lignin biosynthetic proteins (**b**) were predicted from the protein search against the STRING database

To obtain insight into the interaction of 42 candidate proteins and four regulators involved in the proposed model of fiber growth, we carried out bioinformatics analysis to predict the protein–protein interactions by searching against the STRING database [26]. Ultimately, 28 interacting pairs involved in 15 proteins were identified, which constituted the two following interaction networks: one comprised of eight cellulose biosynthetic proteins (including seven CesAs and one CesA-interactive protein whole_GLEAN_10019557) and another comprised of seven lignin-biosynthetic proteins (Fig. 4).

## Discussion

Currently, there were many enzyme-encoding genes associated with cellulose and lignin biosynthesis have been reported [5, 7, 8, 36], and RNA sequencing has identified scores of differentially expressed genes and non-coding RNAs from the fiber developmental barks of ramie [7, 9, 10]. However, a comprehensive understanding for the mechanism underlying fiber growth remains largely unknown in this fiber crop. Fibers in the barks of TPS and MPS are under different growth stage, and thereby, were frequently used as an ideal material for researching the fiber growth in many previous studies [7, 9, 10, 37, 38]. In this study, we identified scores of fiber growth-related genes and determined their putative role in the secondary wall biosynthesis of fibers using orthologous analysis. In recent, at least five ramie genes had been identified to be involved in the biosynthesis of secondary walls, and their overexpression could cause a change of fiber growth in transgenic *Arabidopsi*s [37–40], indicating that there is a conserved mechanism in the biosynthesis of secondary walls between the fibers of *Arabidopsis* and ramie, and therefore, it is feasible to analyse the mechanism underlying the secondary wall biosynthesis by the orthologous analysis. Consequently, this study proposed a model for the secondary wall biosynthesis of fibers in ramie firstly, based on the orthologous analysis. The CesAs are pivotal enzyme for the biosynthesis of cellulose, and seven CesAs were included in this proposed model; however, four of which displayed upregulated expression, and three have a downregulated expression in the MPS. Furthermore, bioinformatics interaction analysis indicated a closer interaction among CesAs with the same direction of expression changes than CesAs with those that were different. Collectively, these results suggested that the function of ramie CesAs in the fiber growth probably have a divergency.

Past studies on model plants have indicated that a NAC-MYB-based transcriptional regulatory network is essential for the modulation of the secondary wall^1^. In *Arabidopsis*, at least 16 MYB proteins and 13 NAC proteins are involved in this regulatory network [1]. Recently, several transcriptional regulators have been identified to be involved in the fiber growth of ramie [37–40], including two NAC proteins Bnt03G004997 and Bnt08G012573 that are the orthologs of *Arabidopsis* VND4/VND5 and NST1/NST2, respectively. Overexpression of ramie MYB gene whole_GLEAN_10015497 caused a significant increase in the fiber number, as well a distinct thickening in the secondary wall, in the transgenic *Arabidopsis* [37]. However, in this study, only one NAC and one MYB protein were identified with expressed change in the fiber developmental barks. Unlike the enzymes, transcription factors generally have a relatively low abundance that results in a low sensitivity in protein detection, which probably is an important reason for the limited transcriptional regulators identified in this study.

KNOX proteins play a negative role in fiber growth by decreasing the deposition of secondary cellular walls [41–44]. Loss of the function of KNAT1 and STM leads to a reduction in the formation of xylem fibers [35], suggesting that KNOX protein exerts a complex function in the regulation of fiber growth. This study identified a ramie KNOX protein whole_GLEAN_10029667 that showed differential expression in the MPS and TPS of ramie. A recent study has performed a function analysis for *whole_GLEAN_10029667*, and its overexpression could significantly reduce the fiber number of transgenic *Arabidopsis.* Interestingly, the expression of *whole_GLEAN_10029667* underwent an epigenetic regulation by circular RNAs [10], and the function of this KNOX protein also was to precisely regulate by phosphorylation modification [38]. Therefore, our protein expressed result further suggest that whole_GLEAN_10029667 plays a central role in the regulation of fiber growth. Taken together, this study provides proteomic insights into fiber growth in ramie, and the identification of many candidates associated with fiber growth provides important basis for understanding the fiber growth in this crop.

## Conclusion

In this study, we identified a total of 983 proteins with differential expression in the fiber developmental barks of ramie by proteomic analysis. Forty-six differentially expressed proteins were found to be homolog of known secondary wall-biosynthetic proteins of *Arabidopsis*, thereby to be considered as the potential candidate associated with fiber growth of ramie. Finally, a molecular model for the secondary wall biosynthesis of ramie fibers was proposed. Taken together, the identification of fiber growth-related candidates provide important basis for understanding fiber growth in ramie.

## Supplementary Information

**Additional file 1.**

**Additional file 2.**

## Data Availability

The mass spectrometry data from the proteome and PRM analysis have been deposited to the ProteomeXchange Consortium (http://proteomecentral.proteomexchange.org) via the iProX partner repository with the dataset identifier PXD026596 and PXD026598, respectively.
