# Supplementary material for: Identification of proteins associated with bast fiber growth of ramie by differential proteomic analysis
Source: BMC Genomics. 2021 Dec 2;22:865. doi: 10.1186/s12864-021-08195-9 (PMC8638140; doi:10.1186/s12864-021-08195-9)
Supplement: Supplementary file 1 — Additional file 1. [file 12864_2021_8195_MOESM1_ESM.docx]

**Table S2** GO terms significantly enriched by differentially expressed proteins

|  | GO Terms | Description | Fold | P value |
| --- | --- | --- | --- | --- |
| Cellular Component | GO:0030312 | external encapsulating structure | 4.7 | 1.1×10^-5^ |
|  | GO:0005618 | cell wall | 4.7 | 1.1×10^-5^ |
|  | GO:0005576 | extracellular region | 3.8 | 5.3×10^-5^ |
|  | GO:0071944 | cell periphery | 2.8 | 3.6×10^-4^ |
|  | GO:0031224 | intrinsic component of membrane | 1.6 | 7.5×10^-4^ |
|  | GO:0016021 | integral component of membrane | 1.6 | 1.2×10^-3^ |
|  | GO:0048046 | apoplast | 4.1 | 4.3×10^-3^ |
|  | GO:0031226 | intrinsic component of plasma membrane | 9.9 | 1.0×10^-2^ |
| Molecular Function | GO:0016798 | hydrolase activity, acting on glycosyl bonds | 2.3 | 2.8×10^-9^ |
|  | GO:0004601 | peroxidase activity | 3.7 | 5.5×10^-9^ |
|  | GO:0020037 | heme binding | 2.3 | 8.3×10^-8^ |
|  | GO:0046906 | tetrapyrrole binding | 2.2 | 3.5×10^-7^ |
|  | GO:0016209 | antioxidant activity | 3.0 | 4.3×10^-7^ |
|  | GO:0004097 | catechol oxidase activity | 5.4 | 2.6×10^-6^ |
|  | GO:0016757 | transferase activity, transferring glycosyl groups | 1.9 | 1.3×10^-5^ |
|  | GO:0004568 | chitinase activity | 4.3 | 1.9×10^-5^ |
| Biological Process | GO:0042744 | hydrogen peroxide catabolic process | 5.2 | 2.3×10^-10^ |
|  | GO:0044036 | cell wall macromolecule metabolic process | 4.2 | 1.7×10^-7^ |
|  | GO:0006869 | lipid transport | 4.4 | 1.7×10^-6^ |
|  | GO:0016998 | cell wall macromolecule catabolic process | 4.7 | 1.7×10^-5^ |
|  | GO:0006026 | aminoglycan catabolic process | 4.3 | 1.7×10^-5^ |
|  | GO:0046348 | amino sugar catabolic process | 4.3 | 1.7×10^-5^ |
|  | GO:0006030 | chitin metabolic process | 4.3 | 1.7×10^-5^ |
|  | GO:1901071 | glucosamine-containing compound metabolic process | 4.3 | 1.7×10^-5^ |
|  | GO:1901072 | glucosamine-containing compound catabolic process | 4.3 | 1.7×10^-5^ |
|  | GO:0044264 | cellular polysaccharide metabolic process | 2.5 | 3.5×10^-4^ |
|  | GO:0015994 | chlorophyll metabolic process | 5.2 | 7.9×10^-4^ |
|  | GO:0010383 | cell wall polysaccharide metabolic process | 4.4 | 8.4×10^-4^ |
|  | GO:0034637 | cellular carbohydrate biosynthetic process | 2.4 | 9.3×10^-4^ |
|  | GO:0005984 | disaccharide metabolic process | 3.6 | 1.3×10^-3^ |

**Table S3** Candidate proteins for fiber growth of ramie based on the orthologous analysis of Arabidopsis secondary wall-biosynthetic protein

| Protein ID | Direction of expression change | Annotation |
| --- | --- | --- |
| **Cellulose biosynthesis and assembly** | |  |
| whole_GLEAN_10024039 | up | Cellulose synthase IRX1 |
| whole_GLEAN_10007243 | up | Cellulose synthase IRX3 |
| whole_GLEAN_10021497 | up | Cellulose synthase IRX5 |
| whole_GLEAN_10017526 | up | Cellulose synthase |
| whole_GLEAN_10005256 | down | Cellulose synthase |
| whole_GLEAN_10016752 | down | Cellulose synthase |
| whole_GLEAN_10027761 | down | Cellulose synthase |
| whole_GLEAN_10019557 | down | Cellulose Synthase Interactive 1-like isoform CSI1 |
| whole_GLEAN_10011261 | up | Sucrose synthase |
| whole_GLEAN_10020223 | up | Sucrose synthase |
| whole_GLEAN_10017913 | up | Sucrose synthase |
| whole_GLEAN_10025884 | up | Sucrose synthase |
| whole_GLEAN_10011928 | down | Endo-1,4-beta-glucanase KOR |
| whole_GLEAN_10006702 | up | Endo-1,4-beta-glucanase KOR |
| whole_GLEAN_10018858 | up | Chitinase-like protein 2 (CTL2) |
| **Lignin biosynthesis** | |  |
| whole_GLEAN_10023841 | up | cinnamoyl-CoA reductase CCR |
| whole_GLEAN_10016789 | up | cinnamoyl-CoA reductase CCR |
| whole_GLEAN_10023293 | up | cinnamoyl-CoA reductase CCR |
| whole_GLEAN_10016783 | up | Cinnamoyl-CoA reductase CCR |
| whole_GLEAN_10005908 | up | Cinnamyl alcohol dehydrogenase CAD |
| whole_GLEAN_10028460 | up | 4-coumarate-CoA ligase 4CL |
| whole_GLEAN_10023365 | up | 4-coumarate--CoA ligase 4CL |
| whole_GLEAN_10026637 | down | caffeoyl-CoA O-methyltransferase CCoAOMT |
| whole_GLEAN_10011593 | down | cinnamate-4-hydroxylase C4H |
| whole_GLEAN_10024942 | up | caffeic acid 3-O-methyltransferase COMT |
| whole_GLEAN_10022572 | up | caffeic acid 3-O-methyltransferase COMT |
| whole_GLEAN_10024940 | up | caffeic acid 3-O-methyltransferase COMT |
| whole_GLEAN_10017970 | up | caffeic acid 3-O-methyltransferase COMT |
| whole_GLEAN_10024941 | up | caffeic acid 3-O-methyltransferase COMT |
| whole_GLEAN_10006818 | up | Lignin-forming anionic peroxidase (PRX) |
| whole_GLEAN_10022017 | up | peroxidase 2 (PRX) |
| whole_GLEAN_10022020 | up | peroxidase 2 (PRX) |
| whole_GLEAN_10021805 | up | Peroxidase 72 (PRX) |
| whole_GLEAN_10010052 | up | phenylalanine ammonia-lyase (PAL) |
| whole_GLEAN_10022662 | down | Phenylalanine ammonia-lyase (PAL) |
| **Xylan biosynthesis** |  |  |
| whole_GLEAN_10018767 | up | Xylosyltransferase IRX10L |
| whole_GLEAN_10008444 | down | protein IRX15-LIKE (IRX15L) |
| whole_GLEAN_10024150 | down | Reduced Wall Acrtylation 3 (RWA3) |
| **Glucomannan biosynthesis** | |  |
| whole_GLEAN_10016190 | up | Glucomannan 4-beta-mannosyltransferase 9 CslA9 |
| whole_GLEAN_10005227 | down | Glucomannan 4-beta-mannosyltransferase 2 CslA2 |
| whole_GLEAN_10022522 | down | Glucomannan 4-beta-mannosyltransferase 9 CslA9 |
| **Deposit of secondary walls** | |  |
| whole_GLEAN_10013214 | up | 65-kDa microtubule-associated protein 8 (MAP65–8) |
| **Regulatory proteins** |  |  |
| whole_GLEAN_10023515 | up | Protein Walls Are Thin 1 (WAT1) |
| whole_GLEAN_10019634 | up | MYB protein |
| whole_GLEAN_10007831 | up | NAC protein |
| whole_GLEAN_10029667 | up | Knotted-like homeobox protein |


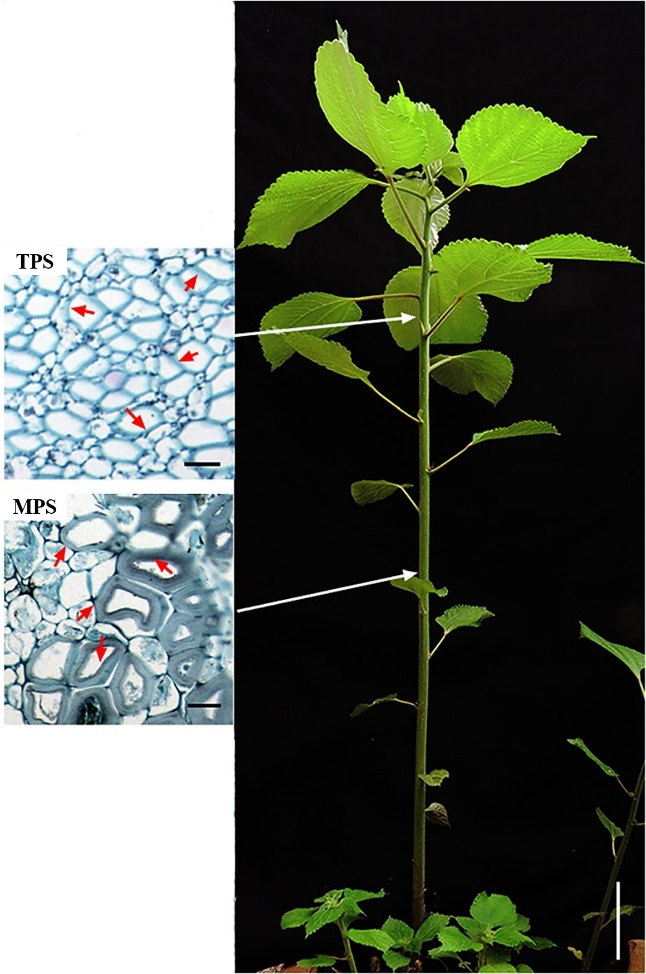


**Fig S1** Microscopic observation of fiber cells from the stem barks of ramie (published by Chen et al. 2014, BMC Genomics, 15:919). The barks from the top part of stem (TPS) and from the middle part of stem (MPS) where the secondary cellular walls have not initiated the growth and was thickening, respectively, were used for proteome analysis in this study.


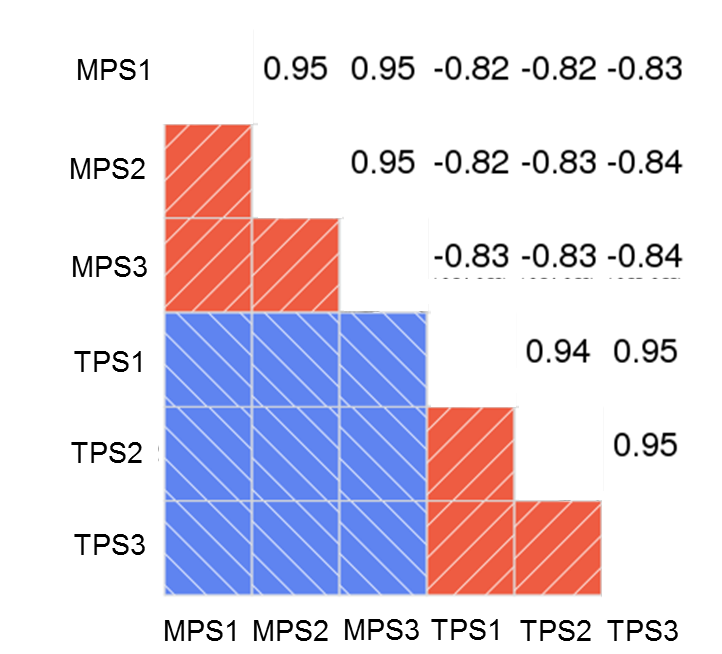


**Fig S2** Evaluation of the repeatability among replicates based on the Pearson correlation analysis. The number in the graph indicated the correlation coefficient, and the red and blue table represented the positive and negative correlation. TPS and MPS in figure indicated the proteome for the barks from the top part of stem and from the middle part of stem, respectively.
